# A pilot study shows the positive effects of continuous airway pressure for treating hypernasal speech in children with infantile-onset Pompe disease

**DOI:** 10.1038/s41598-021-97877-1

**Published:** 2021-09-22

**Authors:** Yin-Ting Zeng, Wen-Yu Liu, Pao-Chuan Torng, Wuh-Liang Hwu, Ni-Chung Lee, Chun-Yi Lin, Yin-Hsiu Chien

**Affiliations:** 1 Department of Medical Genetics, National Taiwan University Hospital, Taipei, Taiwan; 2 Department of Physical Therapy and Graduate Institute of Rehabilitation Science, College of Medicine, Chang Gung University, Taoyuan, Taiwan; 3 Present Address: Department of Audiology and Speech-Language Pathology, Asia University, Taichung, Taiwan; 4 Department of Pediatrics, National Taiwan University College of Medicine, Taipei, Taiwan; 5 Department of Speech-Language Pathology and Audiology, National Taipei University of Nursing and Health Sciences, Taipei, Taiwan

**Keywords:** Neurogenesis, Neurological disorders, Oral diseases, Genetic counselling, Rehabilitation, Diseases, Health care, Neurology, Pathogenesis, Signs and symptoms

## Abstract

Children with infantile-onset Pompe disease (IOPD) demonstrate hypernasality. This study aimed to evaluate whether continuous positive airway pressure (CPAP) training may reduce hypernasality in children with IOPD. Five children with IOPD were enrolled in a single-subject experimental design of type A-B-A′. The intervention comprised an 8-week, 6-day-per-week regimen of CPAP training at home. Participants continued traditional speech therapy once per week throughout the 24-week study duration. The outcome measurements included the degree of hypernasality (DH), the percentage of consonants correct (PCC), and the speech intelligibility score (SIS). C-statistic analysis with an α of 0.05 was used along with visual analysis to assess speech changes. Three patients completed the study. During the CPAP training phase, the DH, PCC, and SIS were significantly improved compared with the baseline (*p* < 0.05). At the follow-up phase, both DH and SIS were improved compared with the baseline (*p* < 0.05), but the PCC had returned to the baseline level. CPAP training demonstrated effectiveness in reducing nasal sounds in IOPD patients. Further studies training younger children with normal hearing may help elucidate the persistence of the effects in children with IOPD.

## Introduction

Pompe disease is a lysosomal disorder in which a deficiency in acid α-glucosidase (GAA) causes intralysosomal accumulation of glycogen in all tissues, most notably in cardiac, skeletal, and smooth muscle cells^1^. Enzyme replacement therapy (ERT) with recombinant human GAA (rhGAA; Myozyme^®^, alglucosidase-α) is the only treatment currently available. ERT effectively reverses cardiomyopathy, improves motor development, and improves overall survival^2^. However, significantly disabling speech disorders, including hypernasality, articulation disorder, and impaired speech intelligibility, frequently occur in children with infantile-onset Pompe disease (IOPD), even with early treatment or high-dosage ERT^3–6^.

Speech disorders in children with IOPD are likely due to velopharyngeal incompetence^4^, which can be caused by the impairment of muscles (including the levator palatini muscles, palatoglossus muscles, and palatopharyngeus muscle) or nerves (including the vagus nerve, facial nerve, and glossopharyngeal nerve). Each movement results from the synergistic activity of several muscles; for example, the levator veli palatini serves as a sling to pull the velum up and back toward the posterior pharyngeal wall; the palatoglossus brings the velum down for nasal consonants; the palatopharyngeus narrows the pharynx by pulling the lateral pharyngeal walls upward and medially. The movement patterns of the velopharyngeal muscles are complex^7^. Although muscle impairment in IOPD can be rescued by ERT^8^, the effect may be inadequate or incomplete^9^ and may not reverse neuronal involvement in Pompe disease^10^.

Continuous positive airway pressure (CPAP), first introduced by Kuehn, can provide resistance training to strengthen velopharyngeal closure muscles^11^. Previous research demonstrated that CPAP training effectively reduces hypernasality^11–15^ and strengthens the muscles involved in velopharyngeal closure^15,16^. To date, the subjects in these studies have included children with cranial abnormalities^11–13^, adults with brain injuries^11,14^, and healthy participants^12,15^. There have been no studies using CPAP as a treatment for hypernasality in children with progressive deterioration in muscle/neuronal function, such as patients with IOPD or other neuromuscular disorders.

In this study, we aimed to train the muscles involved in velopharyngeal closure in children with IOPD using CPAP training to determine whether hypernasality, articulation disorders, and speech intelligibility could be improved further with this method than with traditional speech therapy alone.

## Methods

The study was a single-subject experimental design replicated across subjects. The Chang Gung Medical Foundation Institutional Review Board approved all experimental protocols (study ID Number 201401043RINA). All experiments were performed according to the relevant guidelines and regulations. Informed consent was obtained from the parents, and the subjects provided assent for participation.

### Participants

Children with early-stage, treated IOPD were enrolled. All of them had been diagnosed by both enzyme assays and mutation analysis at National Taiwan University Hospital, had received ERT since birth^2,17^, and had been referred to a speech and language pathologist (SLP) due to speech disorders. Children were required to be older than 4 years old to be enrolled in this study. Speech therapy and assessment were scheduled once per week for 24 weeks. The outcomes of children who completed all of the evaluations in the 24-week study are reported.

### Design

This study used a single-subject experimental design (SSED) of type A-B-A′, to determine whether CPAP training could improve hypernasality, articulation, and speech intelligibility in children with IOPD. Each participant received weekly speech therapy for 8 weeks as the baseline phase (A) and continued this weekly speech therapy for the 8-week CPAP training phase (B) and the subsequent 8 weeks of follow-up (A′). In the CPAP training phase (B), the child additionally received CPAP training 6 days per week for 8 weeks. Each session of speech therapy was recorded and scored at the end of the study.

### CPAP training

**Figure 1:**
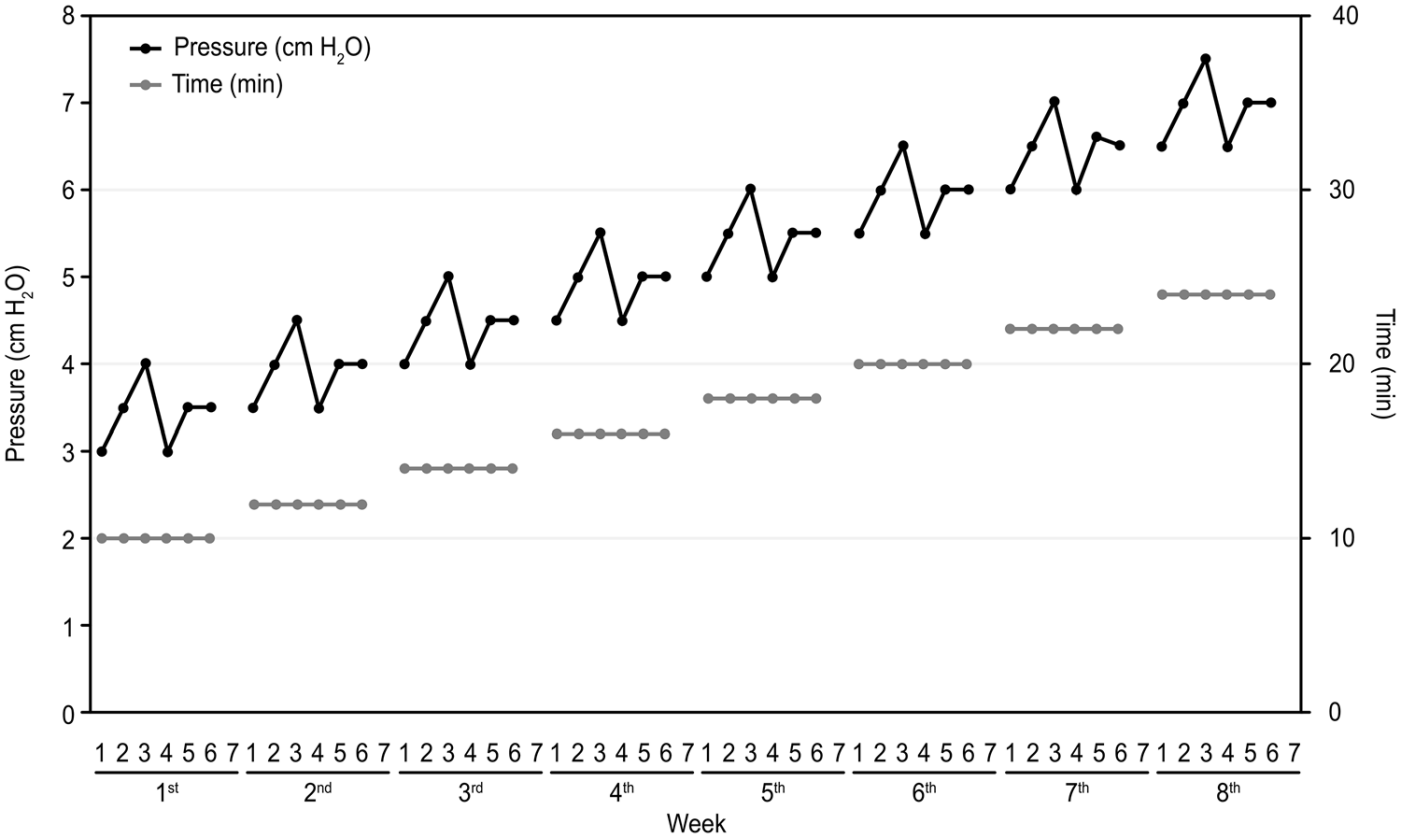
The home CPAP training program. The horizontal axis shows the 8 weeks of the training phase subdivided into 7 days per week. The vertical axis on the left shows the value of the CPAP, which is represented by the black dots and black lines. The vertical axis on the right shows the CPAP usage time, which is represented by the gray dots and gray lines.

All children participated in an 8-week, 6-day-per-week regimen of caregiver-administered CPAP training using standard equipment (DeVilbiss IntelliPAP^®^ AutoAdjust^®^ System, DV54D series, USA) at home with their parents’ assistance. The protocol was similar to that used in a previous study^13^. The researcher increased the pressure in nonlinear increments from 3 cmH_2_O to 7.5 cmH_2_O (Fig. 1), 1 cmH_2_O less than the pressure used in the literature. The duration of the training was increased slightly every week from 10 to 24 min in linear steps throughout the program. The nasal masks were available in different sizes to best fit the various subjects and were worn as previously described.

During each CPAP training session, the children were asked to produce a set of 75 speech samples that consisted of specially designed utterances of the type VNCV, where V represents a vowel, N a nasal consonant, and C a pressure consonant (any stop, fricative, or affricate). They were also directed to speak 10 short sentences from a children's storybook^13^, such as ‘All the neighbors had a cold.’ All VNCV samples were phonologically permissible in Mandarin. All subjects read those words by themselves except Subject 3, who repeated the words after the mother read them aloud.

### Outcome assessments

#### Recordings equipment

Recordings were performed with a handheld digital audio recorder (Zoom H5 Handy Recorder, ZOOM, NY, USA) with recordings stored as 16-bit/44.1-kHz waveforms. All recordings were performed using a standardized setup in a quiet room on a one-to-one basis to obtain voice samples. The recorder was placed 10 cm away from the subjects’ lips to reduce unnecessary background noise.

#### Speech evaluation

Speech assessments were performed once per week until week 24. The SLP scored each session on the spot in addition to recording the speech samples for further evaluation and scoring. During the speech assessment, children were instructed to name 20 randomly presented pictures in the ‘word-level consonant articulation’ subtest of a Mandarin articulation test^18^.

All the scores were generated by a senior SLP with more than 15 years of clinical experience who scored the speech samples on the spot (OTS) and re-evaluated three randomly selected samples from each patient (a total of nine speech samples) at the end of the study to assess intrarater reliability. Speech samples were scored according to perceptual rating scales covering resonance (evaluated by degree of hypernasality, DH), articulation (evaluated by the percentage of consonants correct, PCC), and overall intelligibility (evaluated by the speech intelligibility score, SIS). The SLP also provided speech therapy for all children and instructions for their parents. A second senior SLP (who had practiced child speech and language therapy full time for 5 years) also evaluated the nine selected speech samples to assess interrater reliability. The intraclass correlation coefficient (ICC) of the scores was calculated to assess reliability.

The scoring was the same as previously described^4^. Briefly, the degree of hypernasality (DH) was rated from 1 to 7 (1 for no hypernasality and 7 for the most severe hypernasality), the speech intelligibility score (SIS) was assessed on a scale from 1 to 5 (1 for complete unintelligibility and 5 for complete intelligibility), and the percentage of consonants correct (PCC) was scored and rated from 0 to 100% (normal children over 3 years old should have a PCC of more than 85%).

### Data analysis

**Figure 2:**
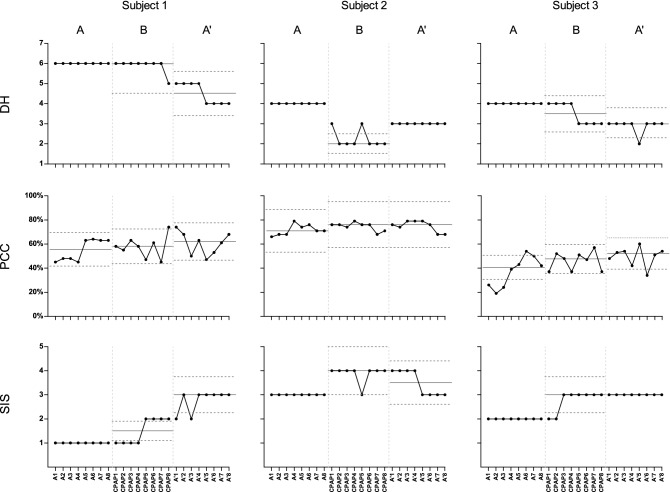
Graphic display of the degree of hypernasality (DH), the percentage of consonants correct (PCC), and the speech intelligibility score (SIS) of Subjects 1-3 during the study. The horizontal axis shows eight sessions in each condition: A (baseline), B (CPAP training), and A′ (follow-up). The top graphs show DH data, the middle graphs show PCC data, and the bottom graphs show SIS data. The thin solid line is the median value of each phase, and the stability envelope for each phase is between the dotted lines (within 25% of the median).

DH, PCC, and SIS data were displayed for visual analysis of graphed data using a single-subject experimental design (Fig. 2). Data were graphed for each participant during the study with the median value (thin solid line) and stability envelope (between the dotted lines) for conditions A (baseline), B (CPAP training), and A′ (follow-up). C-statistic analysis (Table 3) with a 1-tailed α of 0.05 (z ≥ 1.645) was performed in Excel 2016 to assess score changes between the baseline, CPAP training, and follow-up phases^19^.

## Results

### Subjects

Five children (2 males and 3 females) were enrolled in this study. They were at a mean age of 8 y 11 m (age 5 y 10 m–9 y 11 m, mean = 8.4, SD = 1.55). ERT was started at 5–89 days of age (Table 1). They all presented with hypertrophic cardiomyopathy upon diagnosis, and their genotypes have been reported previously^17^. All the children began attending normal schools at an appropriate age.

**Table 1:** Participant demographics.

No	Gender	GA (weeks)	ERT age (days)	Age (y;m)	Middle ear function	Type of hearing loss	Hearing aids	Cognition	Language	Speech therapy
1	F	39	26	9;11	Negative pressure	C (bilateral)	+	Borderline	Borderline	1 session/week since 6.5 years old
2	M	39	17	8;5	PE-tube	Mix (bilateral)	+	Normal	Normal	1 session/week since 3.5 years old
3	F	38	5	5;10	Negative pressure	S (bilateral)	−	Normal	Normal	1 session/week since 5.5 years old
4	M	40	89	9;0	Negative pressure combined TM perforation	Mix (bilateral)	−	Normal	Normal	Unregular ST since 4 years old
5	M	40	29	8;11	TM perforation	C (bilateral)	−	Normal	Normal	1 session/week since 3.5 years old

GA: gestational age; ERT: enzyme replacement therapy; PE-tube: pressure equalizing tube; TM: tympanic membrane; C: conductive hearing loss; S: sensorineural hearing loss; Mix: Combined characteristics of conductive and sensorineural hearing loss.

All of the children had abnormal middle ear function and presented with conductive, sensorineural, or mixed bilateral hearing loss (Table 1). Even Subject 2, who had undergone PE-tube surgery, had hearing thresholds above the normal limits. All children had more severe hearing loss in the high-frequency range (2–8 kHz) than in any other range, and Subjects 1 and 2 used hearing aids. According to the prior year’s evaluation, 3 of the 5 children had normal cognition, and 4 of the 5 children had normal language development (only Subjects 1 and 4 had borderline cognitive performance, and only Subject 1 had a comprehensive language development delay) (Table 2). However, Subjects 3 and 4 had delayed language development before 3 years of age, borderline language development at 3–4 years of age, and normal language development at 6 years of age.

**Table 2:** Results of cognition and language standardized test.

No	Gender	Age (y;m)	Wechsler preschool and primary scale of intelligence-revised (WPPSI-R)			Preschool language scale-revised, PLS-R/language scale-revised, LS-R			
			Verbal IQ	Percentile	Performance Level	Perceptive language (percentile)	Expressive language (percentile)	Full language (percentile)	Performance Level
1	F	9;11	80	9	Borderline	1	10	3	Abnormal
2	M	8;5	111	77	Upper-middle	93	95	99	Normal
3	F	5;10	91	27	Middle Lower	39	49	52	Normal
4	M	9;0	75	5	Borderline	10	19	13	Normal
5	M	8;11	105	63	Medium	49	80	68	Normal

### Outcome assessments

Three children (Subjects 1, 2, and 3) completed all outcome assessments for the full 24 weeks. In the first week of CPAP training, Subjects 1 and 3 experienced copious mucus leaks from the nose and the mouth while wearing their masks; accordingly, the first week of training focused on acclimation to CPAP and they did not start to practice their speech with the device until the second week. Therefore, CPAP training was implemented from the second to the ninth week in those children. Subject 2 stopped CPAP training in the third week due to the emergence of influenza-like symptoms. He then resumed the training and completed weeks 4 through 9 according to the original schedule. Subjects 4 and 5 did not complete the study. Subject 4 came to the hospital for fewer than one-third of the sessions, and Subject 5 merely used the CPAP device at home for the first week. No major adverse effects were reported during the study. The nasal mask of the CPAP apparatus caused discomfort in Subject 3 due to the edges of the mask pressing into her face. We changed the type and size of her nasal mask, and she did not complain afterwards.

A total of 72 speech samples, each containing 20 words, were successfully obtained from these 3 children. The ICC for interrater reliability was 0.965 for DH, 0.991 for PCC, and 0.978 for SIS. The ICC for intrarater reliability was 0.972 for DH, 0.990 for PCC, and 0.980 for SIS.

### Degree of hypernasality (DH)

All three subjects showed hypernasality at baseline, with 6, 4, and 4 points on the 7-point scale (Table 3, Fig. 2), and the condition was stationary (Z ≒ 0, all *p* in phase A ≥ 0.05).

**Table 3:** Data of 3 components of speech for all of the 3 participants between the baseline, continuous positive airway pressure (CPAP) training, and follow-up phases by C statistic analysis.

	Phase A					Phase CPAP					Phase A′					Compared Phase A-CPAP					Compared Phase A-A′				
	${\overline{\text{X}}}$	SD	VA	Z	p	${\overline{\text{X}}}$	SD	VA	Z	p	${\overline{\text{X}}}$	SD	VA	Z	p	${\overline{\text{X}}}$	SD	VA	Z	p	${\overline{\text{X}}}$	SD	VA	Z	p
**Subject 1**																									
DH	6.00	0.00	0	≒ 0	0.5000	5.88	0.35	−	2.37	0.0080**	4.50	0.53	−	4.21	0.0000**	5.94	0.25	−	2.46	0.0069**	5.25	0.86	−	5.02	0.0000**
PCC	0.55	0.09	+	2.41	0.0080**	0.58	0.09	+	9.88	0.0000**	0.61	0.10	−	2.74	0.0031**	0.56	0.09	+	3.68	0.0001**	0.58	0.09	+	0.23	0.4090
SIS	1.00	0.00	0	≒ 0	0.5000	1.50	0.53	+	1.40	0.0800	2.75	0.46	+	5.94	0.0000**	1.25	0.45	+	1.86	0.0314*	1.88	0.96	+	1.39	0.0823
**Subject 2**																									
DH	4.00	0.00	0	≒ 0	0.5000	2.25	0.46	−	4.86	0.0000**	3.00	0.00	0	≒ 0	0.5000	4.13	1.96	−	1.67	0.0475*	4.50	1.55	−	2.18	0.0146*
PCC	0.72	0.04	+	4.81	0.0000**	0.75	0.03	0	0.32	0.3740	0.75	0.05	−	5.11	0.0000**	0.73	0.04	+	4.18	0.0000**	0.73	0.05	0	1.09	0.1379
SIS	3.00	0.00	0	≒ 0	0.5000	3.88	0.35	+	1.40	0.0808	3.50	0.53	−	1.40	0.0808	3.44	0.51	+	3.30	0.0005**	3.25	0.45	+	2.42	0.0078**
**Subject 3**																									
DH	4.00	0.00	0	≒0	0.5000	3.50	0.53	−	3.27	0.0005**	2.88	0.35	−	10.46	0.0000**	4.75	1.34	−	2.88	0.0020**	4.44	1.63	−	1.97	0.0244*
PCC	0.37	0.13	0	1.61	0.0537	0.46	0.08	+	8.50	0.0000**	0.49	0.08	0	13.46	0.5000	0.41	0.11	+	0.49	0.3121	0.43	0.12	+	0.35	0.3632
SIS	2.00	0.00	0	≒ 0	0.5000	2.75	0.46	+	1.98	0.0239*	3.00	0.00	0	≒ 0	0.5000	2.38	0.50	+	3.96	0.0000**	2.50	0.52	+	3.63	0.0001**

Phase A: baseline phase; Phase CPAP: CPAP training phase; Phase A′: follow up phase; Compared Phase A-CPAP: compared baseline phase and CPAP training phase; Compared Phase A-A′: compared baseline phase and follow up phase; HN: Hypernasality; PCC: Percentage of Consonants Correct; SIS: Speech Intelligibility Score; VA: Visual analysis, 0: no difference, -: negative change, +: positive change.

**p < 0.01, very significant difference; *p < 0.05, significant difference.

During CPAP training, all subjects showed significant improvement in DH relative to CPAP initiation (Table 3, Phase CPAP) and baseline (Table 3, Phases A and CPAP Compared).

After the cessation of CPAP training (A′, follow-up phase), the DH improved in the maintenance phase for both Subjects 1 and 3 (Table 3, Fig. 2, Phase A′: *p* < 0.0001 in both Subjects 1 and 3). Visual analysis (VA) by contrasting the phases of the A-B-A′ design showed that the stability envelope for conditions A and B in DH underwent a negative change (improved) in the three subjects. The DH did not improve in the follow-up phase in Subject 2 (Table 3, *p* = 0.5000), demonstrating stationarity at 3 points (Fig. 2), and was lower than the value in the baseline phase and higher than the value in the CPAP phase. DH was maintained at 6 points for 6 weeks in Subject 1 and at 3 points in Subject 2 and Subject 3, indicating a persistent effect even after the cessation of CPAP training (*p* < 0.001 for Subject 1, 0.0146 for Subject 2, and 0.0244 for Subject 3) (Table 3).

### Percentage of consonants correct (PCC)

The three subjects had very different PCCs in the baseline phase, both in the mean value and in the degree of variation (Fig. 2). In all patients, the median line of the PCC was elevated in conditions B and A′ (Fig. 2). Nevertheless, when the three periods were compared using C-statistic analysis (Table 3), only Subject 1 showed an improvement in the PCC after CPAP training and during the follow-up period. The PCC of Subject 1 was at its lowest (45%) upon study initiation (point A1) and then continued to increase in the baseline period. Moreover, PCC increased during CPAP training and reached peak values at the end of CPAP training (point CPAP8) and the start of the maintenance phase (point A′1) (0.74 and 0.74, respectively).

### Speech intelligibility score (SIS)

In all patients, the median line of the SIS was elevated in conditions B and A′ (Fig. 2). At baseline, Subject 2 outperformed Subjects 1 and 3, demonstrating the best intelligibility score. Although Subject 1 had a better PCC than Subject 3, their SIS suggested very poor intelligibility. After CPAP training, all three subjects showed improved SISs; the effect persisted in Subject 1 and Subject 3 but lasted only 3 weeks in Subject 2.

The differences between the 3 phases of this study were compared by C-statistic analysis (Table 3). During the CPAP training phase, the DH, PCC, and SIS values of all subjects except the PCC of Subject 3 were significantly better than the corresponding baseline values. In the follow-up phase, the DHs of all three subjects and the SISs of 2 of the three were significantly improved from the corresponding baseline values. While only Subject 1 showed an improvement in the PCC after CPAP training and during the follow-up period, the PCCs of the other 2 subjects showed no statistically significant difference from the baseline levels.

## Discussion

In this study, CPAP training effectively reduced hypernasality and improved overall speech intelligibility in children with IOPD. The results were similar to those of previous studies that used CPAP training^11–15^; however, subjects in those studies did not have progressive muscle disorders. Our data were the first to demonstrate that CPAP training could effectively improve speech function even in the context of progressively deteriorating neuromuscular disorders and that the effect could persist even 2 months after the cessation of training.

Hypernasality, which results from a failure of the velopharyngeal orifice to close, is common in children with primary myopathies such as infantile-onset Pompe disease. Speech therapies, whether they are verbal methods such as the Lee Silverman Voice Treatment^20,21^ or nonverbal exercise methods^21,22^, provide limited therapeutic effects on hypernasality, as shown by our previous attempts in children with IOPD^4^. A palatal lift prosthesis may be necessary for these patients, but no study has demonstrated the effectiveness of such prostheses in improving the speech function of children with IOPD. In addition, surgical intervention such as adenotonsillectomy may worsen hypernasality and should be avoided^23^. CPAP training is safe for children with IOPD and should be the first intervention considered for reducing their hypernasality and improving their speech intelligibility if the availability of those specialized equipment for CPAP training is not an issue.

Mechanistically, the benefit of CPAP training in hypernasality may be due to the effects of resistance training on the velopharynx. During training, the levator muscle exerts increased force to overcome the resistance imposed by the increased air pressure from the CPAP device as the user attempts to achieve velopharyngeal closure; this response could eventually result in increased muscle strength, increased completeness of velopharyngeal closure, and reduced hypernasality^12^. In addition, exercise such as CPAP training involves a regimen with many repetitions of velar elevation^11,14,15,24–27^, thus improving muscle endurance.

On the other hand, it does not show statistically significant changes in PCC for any subject when A-A′ phases are compared, and 2/3 subjects demonstrated statistically significant improvement in PCC when A-CPAP phases are compared (Table 3). However, perhaps the lack of impact on articulation is not surprising considering CPAP training targeted the muscles involved in velopharyngeal closure but not other muscles involved in articulation. Additionally, the persistence of compensatory misarticulations is common in other conditions such as cleft palate after surgical repair. It may be that improvement in hypernasality exhibited with CPAP may allow for greater success with more traditional speech therapy approaches to improve speech sound production.

CPAP may exacerbate middle ear disease due to the increased pressure produced^12^. Since all the children in our study had abnormal middle ear function, we started the training at the lowest CPAP pressure, which was 1 cmH_2_O lower than the initial pressure specified in the literature^13^. None of the subjects with PE-tube implants, negative pressure and/or TM perforation presented with acute middle ear disease or worsening of the middle ear during CPAP training. However, the improvement in hypernasality was slower in our study than in previous studies. In addition to the initial pressure effect, the underlying myopathy in IOPD patients may necessitate an extended training time^28^.

The initiation of CPAP in infantile-onset Pompe disease patients faces two technical challenges. First, excessive mucus, which probably accumulated in the oronasal cavity for some time due to the patients’ weak facial, oral, and pharyngeal muscles, leaked from the nose and mouth upon CPAP initiation during the first few days. Such secretions need to be cleaned from the device carefully. Second, the nasal mask may cause discomfort due to the edges of the mask pressing into the face. Adjusting the headwear to a comfortable tightness before starting treatment and allowing pauses during CPAP training can help improve patients’ comfort and ensure their compliance with the training program.

There are several limitations that must be addressed when interpreting the results of this study. First, we presented only the results from 3 subjects who had received traditional speech therapies for a long period and could cooperate with CPAP training. The inability of other patients to complete the training implies barriers to applying the training to others. Second, all patients were evaluated by the same unblinded SLP. Therefore, we compared the intrarater reliability and interrater variability to demonstrate objectivity, and we confirmed that the degree of reliability was excellent (all correlation coefficients were above 0.965). Third, we did not include a control group to demonstrate the effect of maturation under normal conditions. The A-B-A′ (baseline-training-follow-up) experimental design demonstrated that speech could be improved after CPAP training. Fourth, other factors, such as an increase in the rhGAA dose^4^, age, hearing ability, and cognitive development^29^, were all possible confounders of the CPAP training effect. We maintained constant rhGAA doses during the study but could enroll only patients with almost complete phonological development and severe hearing loss in the high-frequency range (2–8 kHz), a range coinciding with most Mandarin consonants (for example, /tʂ, tʂh, ɕ, ʦ, ʨ, ʦh, ʨh, p, t, s/). Further studies, such as interventions in younger patients with the better hearing ability or in patients wearing hearing aids for high-frequency hearing loss, are warranted to elucidate the effects of CPAP training, especially for consonant correction.

## Conclusions

This study suggests that speech disorders in children with IOPD improved following CPAP training, especially in terms of hypernasality and speech intelligibility. Even with the cessation of CPAP training, the benefit was largely maintained during an 8-week follow-up period. Further studies with longer follow-up in a larger cohort, including younger subjects with better hearing ability, are warranted to demonstrate the benefit of CPAP training in children with IOPD.

## Abbreviations

IOPD: Infantile-onset Pompe disease
ERT: Enzyme replacement therapy
CPAP: Continuous positive airway pressure
DH: Degree of hypernasality
PCC: Percentage of consonants correct
SIS: Speech intelligibility score
